# State-of-the-Art Metabolomics and Lipidomics in Life Sciences: Methods and Applications

**DOI:** 10.3390/metabo14010008

**Published:** 2023-12-21

**Authors:** Xinyu Liu, Cora Weigert

**Affiliations:** 1 CAS Key Laboratory of Separation Science for Analytical Chemistry, Dalian Institute of Chemical Physics, Chinese Academy of Sciences, Dalian 116023, China; 2 Liaoning Province Key Laboratory of Metabolomics, Dalian 116023, China; 3 Department for Diagnostic Laboratory Medicine, Institute for Clinical Chemistry and Pathobiochemistry, University Hospital Tübingen, 72076 Tüebingen, Germany; 4 Institute for Diabetes Research and Metabolic Diseases, Helmholtz Zentrum München, University of Tübingen, 72076 Tübingen, Germany; 5 German Center for Diabetes Research (DZD), 90451 Nuremberg, Germany

This Special Issue was initiated to celebrate and congratulate Prof. Guowang Xu and Prof. Rainer Lehmann on their long-standing, fruitful Sino-German scientific cooperation and their close friendship. Their interdisciplinary interaction in the field of high-resolution analytical chemistry and life sciences has existed for decades. The first joint publications addressing biomedical topics and the development of new capillary electrophoresis approaches date back to 1998 [1,2]. Two reviews and twelve original research articles were accepted for publication in this Special Issue. The articles address different aspects of life science research, from microbes, plants and animals to humans applying metabolomics or lipidomics, and new biomarkers, protocols, strategies or bioinformatic tools are reported on. 

The Sino-German scientific cooperation between the Dalian Institute of Chemical Physics of the Chinese Academy of Sciences and the University of Tuebingen was originally initiated in the 1970s by Prof. Peichang Lu, the pioneer of chromatography in China, and Prof. Ernst Bayer, a pioneer of gas chromatography in Europe. Since then, a lively, regular scientific exchange between Dalian and Tuebingen developed. In the mid-nineties, Prof. Guowang Xu and Prof. Rainer Lehmann started their joint research activities during Prof. Xu’s two-year research stay in Tuebingen as a Max-Planck-Institute fellow. 

First, as mentioned above, their joint bioanalytical interest was the development of new applications of capillary electrophoresis for the analysis of human body fluids for diagnostic purposes. Later on, high-resolution mass spectrometric profiling in a biomedical context increasingly became the focus of their joint research interest. In particular, the comprehensive investigation of metabolite and lipid profiles by metabolomics and lipidomics analyses has remained a core theme of their cooperation. In addition to various body fluids, other sample materials like biopsies from various tissues, human primary cell culture samples, etc., were analyzed to investigate pathomechanisms and to identify diagnostic biomarkers of metabolic diseases (prediabetes and diabetes) and various cancer diseases. Additionally, multi-omics approaches were applied in systems medicine studies. An important aspect of their work has been addressing sample quality, considering the error-prone preanalytical phase from “bed to bench”, including the identification of a sample quality biomarker allowing analytical (bio)chemists to assess the quality of blood samples without knowledge about the preceding blood collection and blood handling process. Other cornerstones of their joint research activities include the development of new methods and analytical strategies to either facilitate or increase the identification of metabolites or the coverage of metabolomics and lipidomics approaches, as well as the optimization of sample preparation procedures.

In total, more than 50 joint publications in high-ranking international journals like *Diabetes Care*, *Clinical Chemistry*, *Analytical Chemistry*, etc., have been published by this Sino-German collaboration. The research performed through this collaboration has been regularly supported by joint funds from the national science foundation China (NSFC), the German research foundation (DFG), the Humboldt foundation, as well as from the Chinese Academy of Sciences (CAS). The funding has enabled the exchange of many young scientists between Dalian and Tuebingen, which has built a solid base for continuous and successful cooperation between the CAS Key Laboratory of Separation Science for Analytical Chemistry from the DICP in Dalian and the Institute for Clinical Chemistry and Pathobiochemistry at the University Hospital in Tuebingen.

The 14 publications in this Special Issue cover several thematic aspects of the Sino-German scientific cooperation and are briefly highlighted in the following paragraphs in the order of appearance in the Special Issue.

Jinzhi Xu et al. (2023) (contribution 1) identified lipid metabolism reprogramming due to stemness acquisition in pancreatic ductal adenocarcinoma (PDAC) cancer stem cells (CSCs) using lipidomes combined with the transcriptome analysis of PDAC tumor-repopulating cells (TRCs, a novel CSCs model). The results were supported by the analysis of data obtained from PDAC patients from The Cancer Genome Atlas (TCGA) database. The research also highlighted SPHK1 (sphingosine kinases 1) as the important enzyme involved in the up-regulation of sphingolipid metabolism. Hence, SPHK1 may have a relevant role in PDAC CSCs and may be an appropriate therapeutic target candidate.

Yifei Zhan et al. (2023) (contribution 2) discovered a common molecular mechanism of metabolic acidosis and myocardial damage in neonatal pneumonia. Applying UPLC-HRMS-based untargeted metabolomics, a total of 23 and 21 differential metabolites were found in the comparison of serum samples of pneumonia and samples of pneumonia with the two complications, respectively. The 14 identical molecules found in both disease states were found to be related to sphingolipid, porphyrin, and glycerophospholipid metabolisms, which offers valuable information for the rapid and accurate identification, classification, staging and, most probably, disease diagnosis and therapy of complications of neonatal pneumonia.

Tianfu Wei et al. (2023) (contribution 3) integrated multidisciplinary data to provide an in-depth view of the relationships between genes and metabolites. With this strategy, a prediction model of hepatocellular carcinoma based on nucleotide metabolism was developed. A pattern showing the nucleotide metabolism of patients with hepatocellular carcinoma was elucidated, which created new perspectives for the clinical treatment of hepatocellular carcinoma.

Sijia Zheng et al. (2023) (contribution 4) established valid associations of metabolites in human urine with the metabolism of gut microbiota, which serves as a new biomarker profiling strategy in gut microbiome research. Considering that bowel evacuation was a simple and efficient approach to reveal gut microbiota-related metabolites, a non-targeted modifying group-assisted metabolomics approach was used to investigate urine samples collected in two independent experiments at various time points, before and after laxative use, to drastically reduce the gut microbiome. Additionally, fasting over the same time period was performed as a control experiment. Finally, the levels of 331 urinary metabolite ions were significantly affected by the depletion of the fecal microbiome, including 100 with specific modifying groups, 32 of which were structurally elucidated. The applied strategy has the potential to generate a microbiome-associated metabolite map of urine, and presumably other body fluids as well.

Yixuan Guo et al. (2023) (contribution 5) aims to identify metabolic differences in ankylosing spondylitis (AS) patients at different stages of the disease using an untargeted metabolomics approach based on the gas chromatography–mass spectrometry of serum. The findings of the study indicate that patients in acute stage and remission stage have specific metabolic characteristics. In particular, 2–hydroxybutanoate and hexadecanoate had good efficacy with regard to the stage division of AS. This research may contribute to the understanding of the pathogenesis of ankylosing spondylitis and to the staging treatment of this chronic disease.

Xiaojing Jia et al. (2023) (contribution 6) explored the impact of omega-3 polyunsaturated fatty acids (PUFAs) on inflammatory bowel disease (IBD) using Mendelian randomization. Increased genetically predicted eicosapentaenoic acid (EPA) concentrations are associated with decreased IBD risk, mediated through lower linoleic acid and histidine metabolites. To date, limited evidence has supported the effects of total omega-3, α-linolenic acid and docosahexaenoic acid on IBD risk. Robust colocalization in the *fatty acid desaturase 2 (FADS2)* region suggests *FADS2* gene mediation. Overall, the study highlights EPA as the key active component of omega-3 PUFAs in reducing IBD risk and suggests *FADS2* gene involvement, offering insights for targeted intervention strategies.

Liming Gu et al. (2023) (contribution 7) identified a panel of potential plasma metabolic markers for radiation-induced lung injury (RILI) via correlation analysis between the lung tissue and plasma metabolic features and evaluated the radiation injury levels within 5 days following whole-thorax irradiation (WTI) in a rat model. Moreover, the data imply disorders of the urea cycle, intestinal microbiota metabolism and mitochondrial dysfunction. This research unveils metabolic traits associated with WTI, providing new perspectives on potential therapeutic measures.

Ming Yang et al. (2023) (contribution 8) developed a rapid and highly sensitive detection method for volatile organic metabolites (VOMs) in urine based on the integration of high-pressure photoionization mass spectrometry (HPPI-TOFMS) and dynamic purge-injection technique. Nine differential metabolites in the urine samples between breast cancer patients and healthy controls were successfully identified through statistical analysis of the HPPI MS data. The results demonstrate the good sensitivity and specificity of the method and provide a promising avenue for the development of a new non-invasive diagnostic tool for breast cancer.

The work of Shan Zhang et al. (2023) (contribution 9) compared the differences in the sensory features and chemical profiles of the two grades of premium Dianhong congou black tea (DCT) produced in southwest China and identified the correlations of critical non-volatile compounds and flavor characteristics in these DCTs. This study also highlighted the promising perspective of the integration of metabolomics, electronic tongues, chromatic differences and human sensory evaluation in the analysis of food flavor.

Runze Ouyang et al. (2023) (contribution 10) illustrated the gut microbiota-dependent crosstalk between breast milk N-acetylneuraminic acid (Neu5Ac) and infant growth. The research demonstrates the negative association between breast milk Neu5Ac and infant obesity risk. The data show Neu5Ac-related alterations to infant gut microbiota and bile acid metabolism, and similar associations were found in mice colonized with infant-derived microbiota. Finally, this study identified the mediator between breast milk Neu5Ac and the risk of infant obesity.

Jun Zeng et al. (2023) (contribution 11) analyzed the antiallergic activities of two representative dietary polyphenols, curcumin and epigallocatechin gallate (EGCG), and elucidated their effects on the cellular lipidome in the progression of degranulation. This work contributes to further understanding of the molecular mechanism of antigen stimulation and curcumin/EGCG involvement in antianaphylaxis and helps to guide future attempts to use dietary polyphenols in this context.

Xiaoshan Sun et al. (2023) (contribution 12) introduces a novel serum metabolome characterization method employing direct-infusion high-resolution mass spectrometry, thereby addressing limitations in metabolite assignment. Different from conventional database search, this strategy utilizes a reaction network along with mass accuracy and isotopic pattern filters to achieve unequivocal formula assignments. The developed approach proved database-independent and rapid, assigning unique monoisotopic features in the serum. Its merits lie in the comprehensive and reliable formula assignment, exhibiting strong potential for large-scale metabolomics studies.

In a review by Yilan Ding et al. (2023) (contribution 13), the authors conducted an in-depth literature search on specific amino acids as potential biomarkers for the onset and progression of diabetes. They described underlying mechanisms, signaling pathways, and metabolic implications, providing valuable insights into preventive and therapeutic interventions. Additional clinical research and therapeutic strategies targeting distinct amino acids were discussed, aiming to prevent or slow down the progression to type 2 diabetes in the prediabetic stage.

Shuling He et al. (2023) (contribution 14) performed a systematic, comprehensive literature analysis of metabolomics approaches studying diabetic retinopathy. An overview providing insights into relevant metabolites and metabolic pathways was given. A gap in knowledge in the existing literature was detected with respect to data from large-cohort or multicenter studies, as well as data from platforms applying various analytical metabolomics approaches to study diabetic retinopathy. In addition, future metabolomics research directions with regard to diabetic retinopathy are discussed.

From these contributions and also based on the most recent developments in metabolomics research, we can draw the following conclusions:

1. Analytical challenges to investigate metabolomes and lipidomes in life sciences still exist, such as limitations in metabolite and lipid coverage, a still high number of unknowns in non-targeted approaches and further improvements in analytical sensitivity. However, based on the ongoing continuous improvements and new developments in chromatographic and mass spectrometric techniques, an increasing amount of information on metabolomes can be gathered.
2. Metabolomics and lipidomics analyses can be applied to evaluate the important metabolic effects and functions of molecules and nourishments like dietary polyphenols, polyunsaturated fatty acids, etc., to evaluate the interfering effects of drugs and lifestyle on health.
3. In biomedical and disease-related research fields, metabolomics analysis enables us to study metabolic reprogramming, define potential prospective or prognostic diagnostic biomarkers or subclassify patients in precision medicine by metabolite pattern to allow individualized treatment. However, to draw reliable conclusions based on valid results which reflect the situation in the population, samples of large-scale multi-center studies should be investigated. In this context, a big analytical challenge which needs to be solved is the application of very robust, sensitive and highly reproducible metabolomics methodology, suitable for the analysis of thousands of samples with different pre-analytical quality levels. The comparability of data between laboratories is a fundamental requirement, and quantitative metabolomics will become increasingly important.
4. Metabolomics analysis contributes substantially to deepening the understanding of metabolic mechanisms on the cellular level via investigations like isotope labeling experiments to study dynamics of metabolism or via single-cell metabolomics, which has recently become a hot topic in the breakdown of cell heterogenicity in tissues to elucidate cell type-specific metabolic functions.
5. Traditionally, to study tissue metabolism, homogenates are analyzed, leading to the spatial loss of information. Now, to eliminate this limitation, mass spectrometric imaging or laser microdissection instruments are in use, which are increasingly bringing spatial metabolomics into focus. It is foreseeable that in the near future, with improvements in sensitivity, resolution and speed, spatial and single-cell metabolomics will create new perspectives and play a significant role in the fields of life sciences and biomedical research.
6. Finally, it should be emphasized that a person´s health state is influenced by various factors, and the environment will decide a person´s health. Therefore, a recent research direction, i.e., the combinational use of metabolomics and exposomics, is a fascinating new research field and will be one of the key strategies used to shed light on the assessment of risks and causes of disease. Additionally, and highly relevant in this context, multi-omics applications are already well established in the study of complex diseases, especially chronic diseases, and contribute to our understanding of them. However, currently, a significant challenge is the evaluation and interpretation of these data, as well as the integration of these multi-omics data to support clinical decision-making processes. For this purpose, important contributions from the application of artificial intelligence approaches can be expected from interdisciplinary collaborations.
7. In summary, metabolomics and lipidomics are now well established and frequently applied technologies in life sciences and health promotion, but many bottlenecks still need to be broken by intense, interdisciplinary interaction between scientists from different fields, including analytical (bio)chemists, biologists, bioinformaticians and clinicians.
